# Quantifying community resilience based on fluctuations in visits to points-of-interest derived from digital trace data

**DOI:** 10.1098/rsif.2021.0158

**Published:** 2021-04-28

**Authors:** Cristian Podesta, Natalie Coleman, Amir Esmalian, Faxi Yuan, Ali Mostafavi

**Affiliations:** Urban Resilience.AI Lab, Zachry Department of Civil and Environmental Engineering, Texas A&M University, College Station, TX, USA

**Keywords:** location-intelligence data, community resilience, disaster impacts, infrastructure

## Abstract

This research establishes a methodological framework for quantifying community resilience based on fluctuations in a population's activity during a natural disaster. Visits to points-of-interests (POIs) over time serve as a proxy for activities to capture the combined effects of perturbations in lifestyles, the built environment and the status of business. This study used digital trace data related to unique visits to POIs in the Houston metropolitan area during Hurricane Harvey in 2017. Resilience metrics in the form of systemic impact, duration of impact, and general resilience (GR) values were examined for the region along with their spatial distributions. The results show that certain categories, such as religious organizations and building material and supplies dealers had better resilience metrics—low systemic impact, short duration of impact, and high GR. Other categories such as medical facilities and entertainment had worse resilience metrics—high systemic impact, long duration of impact and low GR. Spatial analyses revealed that areas in the community with lower levels of resilience metrics also experienced extensive flooding. This insight demonstrates the validity of the approach proposed in this study for quantifying and analysing data for community resilience patterns using digital trace/location-intelligence data related to population activities. While this study focused on the Houston metropolitan area and only analysed one natural hazard, the same approach could be applied to other communities and disaster contexts. Such resilience metrics bring valuable insight into prioritizing resource allocation in the recovery process.

## 1. Introduction

Communities affected by natural hazards are complex systems of interacting components [1–4]. The complex systems comprise community residents' lifestyles; services provided by the built environment to residents and businesses; and hazards causing perturbations in the built environment, businesses and daily lifestyles of residents [5]. The definition of community resilience [6] varies depending on the field of interest, but it commonly denotes accessibility and distribution of resources, social connections between residents, and degree of preparedness. The concept of community resilience can inform decisions by community leaders and emergency planners to plan for mitigation of and recovery from disaster impacts. To bridge the gap between resilience theory and application, however, data need to be quantified into specific terminology, concepts, and tools to measure the state of the community during and after the disaster.

Existing studies offer various approaches for quantifying data related to community resilience [7,8]. Quantifying community resilience is an integral part of effective response and resource allocation across disaster phases [9–11]. For instance, studies [12–14] focusing on disaster resilience of the built environment and essential business services, such as hospitals [15,16], schools [17], businesses and supply chains [18,19], oil and gas [20] and grocery stores [21,22] have contributed to the awareness of the physical vulnerability, reliability and recovery of the built environment. An understanding of the connectedness of disparate community components during a disaster can inform the development of effective disaster plans. Existing literature related to community resilience, however, has paid relatively little attention to the interactions among populations, business services, and the built environment [23,24]. In fact, the built environment is regarded as a means rather than an end in the resilience assessment [25]. For example, while the absorptive capacity of hospitals is an important dimension in community resilience, a population's access to and demands upon healthcare services during a disaster should also be assessed [26]. This information is particularly important for quantifying the effects and recovery of community segments due to the different levels of accessibility, need and prioritization of such services [27,28].

Another limitation is the dearth of empirical studies measuring community resilience based on data capturing the complex interactions of the nexus of populations, businesses and the built environment. Most existing studies have relied on two data types: surveys [23,28–32] and social media data [33–38]. Surveys present two drawbacks for collection of community resilience data. First, surveys have limited capacity to capture the holistic and dynamic patterns of recovery due to the timing of survey distribution, scale of analysis, and survey respondent memory after the disaster event [39]. Second, the use of surveys for collecting longitudinal and representative data from locations across disaster-affected regions can be challenging and expensive. As a complement to qualitative survey data, researchers have relied upon social media platforms to quantify data related to community resilience. Social media data have been applied to capturing societal disruptions [35], conducting rapid damage assessment [40–42], and sensing the dynamic situation of infrastructure services [43]. Social media data, however, can overlook certain demographic groups based on user preferences [44]. In addition, it can be difficult to track the dynamic spatial behaviours of users, as only 1–2% of Twitter data contains location information [44]. Considering these limitations of survey and social media data, this study has elected to incorporate a different and relatively new approach to quantifying data for community resilience by building upon the current knowledgebase of the accessibility and need for essential services during the disaster setting.

This research uses digital trace data related to visits to points-of-interest (POIs) to capture the dynamic interactions among population lifestyles, built environment and business services. Digital trace data have seen increasing use in urban mobility studies; however, the use of digital trace data in the context of disaster resilience research has been rather limited [33,45–47] used mobile phone data to investigate migration patterns after a cyclone in Bangladesh. Yabe *et al*. [46,48] analysed mobile phone data to reveal not only the population displacement but also recovery patterns in hurricanes and in the COVID-19 pandemic. These findings shed light on community activities; however, the disaster impacts, and recovery durations of essential business sectors for community's preparation, response and recovery (e.g. gasoline stations and medical facilities) still remain under investigated. To bridge this gap, this study utilizes POI visit data (i.e. digital trace data) to capture community resilience indices (i.e. disaster impacts and recovery durations) of essential business sectors.

Advances in location-intelligence technologies have facilitated access to fine-grained POI visit digital trace data. We propose a methodology for determining community resilience by examining the fluctuations of POI visits and quantifying the extent of impacts and recovery. Digital trace data like POI visits provide a holistic view about the status of communities as it captures population impacts, business interruptions and infrastructure disruptions together. This information enables invested stakeholders, such as community officials and disaster managers, to quickly examine and efficiently monitor disaster impacts and recovery [48]. Thus, the research will implement the methodological frameworks with a case study of the impact of the 2017 Hurricane Harvey on the Houston metropolitan area. It will quantify resilience metrics according to POI visits, including the maximum percentage drop of POI visits (systemic impact) and the maximum duration of disruption of POI visits (duration of impact) to generate the general resilience (GR) curves of POI visits. The research will also examine the spatial distribution of the resilience metrics derived from POI visit fluctuations in conjunction with flooded areas of Houston caused by Hurricane Harvey to understand the relationship between potential damage to POIs and accessibility in different areas. In addition, we have included the spatial distribution of flooding and non-flooding inundation areas caused by Hurricane Harvey. This research answers three questions: (1) what is the extent of impact and duration of recovery of the community based on patterns of visits to POIs across different sectors? (2) To what extent do spatial patterns of impacts and recovery vary based on POI visits patterns? (3) What is the relationship between the metrics derived from POI visits patterns and the extent of flood inundation?

## 2. Methodological background

**Figure 1.**
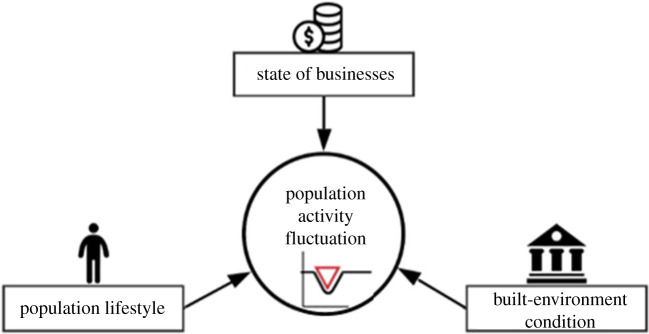
Population activity fluctuations as an indicator of the state-of-community resilience.

The research study examined communities as complex systems and considered POI visits as indicators for capturing the dynamic state of communities. The state of a community is a complex system based on the interactions among population lifestyles, state of businesses and condition of the built environment (figure 1). In this context, population lifestyles refer to the way people live and interact within POI locations in a community. This component accounts for community needs of and expectations from essential services and infrastructure systems. Direct disruptions or demand changes influence the state of the businesses during the hazardous event. The built environment refers to the infrastructure and structures that support community functions. For example, roads provide access to the critical services; disruptions to infrastructure systems may disrupt access to the services. The system is in equilibrium in a normal period without hazards. Perturbations in any component could alter the state of a community, and such changes can be captured based on fluctuations in population activities (e.g. POI visits). The resilience measure obtained based on changes to normal POI visits can be used as a holistic measure of performance (MOP) to quantify community resilience. This data provides insight into the state of community as a complex system and enables the spatial and temporal assessment of the communities in the disaster setting.

### 2.1. Data description

**Figure 2.**
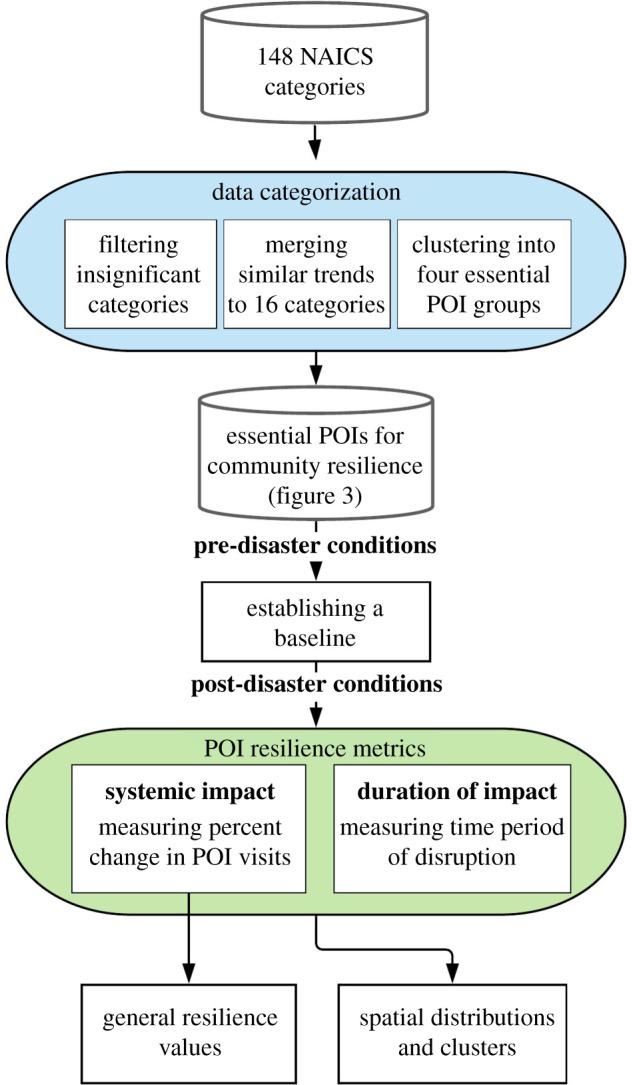
Methodological framework for quantifying data for community resilience using POI visit data.

The research used digital trace data of POI visits in Houston in the context of Hurricane Harvey. Hurricane Harvey made landfall on the Texas Gulf Coast on 25 August 2017, as a category 4 hurricane, inundating Houston with 27–54 inches of rain, shattering most known rainfall records [49]. The data time frame are the period before landfall (before 25 August 2017), during the hurricane (25–31 August 2017), and the weeks of the immediate recovery period (1–30 September 2017). POI visit data were collected from SafeGraph, Inc., which provides the ‘most accurate point-of-interest data and store location geofences for the USA' and collects unique visit instances to physical locations in the community from anonymized cell phone data. Figure 2 shows the methodological framework by which data were quantified and it spatially describes the resilience metrics according to POI visits.

### 2.2. Data categorization

**Figure 3.**
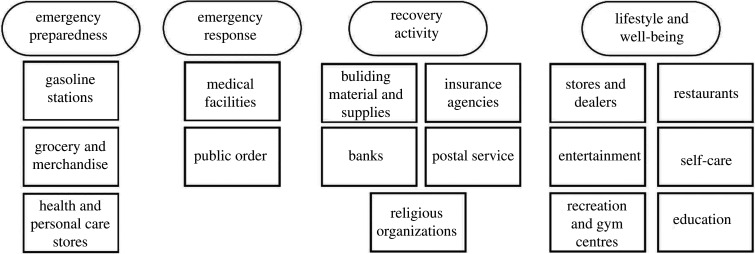
POIs essential for community resilience during disasters.

Each month contained data on 55 537 distinct business entities (POIs) in Houston, including daily visit count information and category type for each POI. Based on the North American Industry Classification System (NAICS), POIs were filtered from 148 specific pre-defined categories into 16 merged categories (see electronic supplementary material). Table 1 shows the selected 16 categories including the reason for selection and reference in the literature. The 16 categories (figure 3) were classified under the four essential POI groups to explain their associations to the disaster impact: POIs essential for (1) emergency preparedness, (2) emergency response, (3) lifestyle and well-being, and (4) recovery activity (figure 3).

**Table 1.** POI groups essential for community resilience.

essential POI groups	categories	reason for selection	citations
emergency preparedness	gasoline stations	gasoline stations provide fuel for evacuation and power generators	[50]
	grocery and merchandise	grocery and merchandise stores provide necessary food and supplies	[21,22]
	health and personal care stores	data show an increase of purchases for preparedness materials before a disaster	[51]
emergency response	medical facilities	resilience frameworks have been created to maintain essential services such as hospitals	[15,16]
	public order	existence of an organizational structure with public order officials serving as first responders to disasters	[52,53]
recovery activity	building material and supplies	priority of debris clearance and immediate rebuilding after disaster	[54,55]
	insurance agencies	data support an increase of purchases of flood insurance for the recovery	[56]
	banks	natural hazards impact the likelihood of a bank default and credit investment, respectively	[57,58]
	postal service	recovery of the USA Postal Service following Hurricane Katrina	[59]
	religious organizations	contribute to social capital indices for disaster resilience and recovery	[31]
lifestyle and well-being	stores and dealers	important references to measure the business and economic recovery	[60,61]
	restaurants		
	entertainment		
	self-care	focus on the long-term well-being impacts as there is a need to return to normalcy	[29,62]
	recreation and gym centres		
	education

## 3. Data analysis

### 3.1. Establishing the baseline for pre-disaster conditions

To understand pre-disaster trends in POI visits, we established a baseline for each of the 16 categories to serve as a community steady state. POI visits during the first three weeks of August 2017 were used to calculate the baseline for all categories except for the education category. Schools were just reopening in mid-to-late August in the USA, resulting in a steady increase in POI visits throughout this period. Instead, the last three weeks of October 2017 were used for the education baseline to obtain a steady trend. To calculate the baseline, an average of the number of visits on each day over the three selected weeks was calculated for every POI category individually, resulting in 16 category-specific baselines.

### 3.2. Calculating per cent change from the baseline

The per cent change indicates the increase or decrease from the baseline of daily visits for each of the 16 major POI categories. In equation (4.1), the individual daily visit counts for each category were used as the daily value, and the baseline points were used as the baseline value. When determining the actual per cent change values throughout the period, each day was compared to its corresponding baseline average. For instance, each Sunday was compared to the Sunday baseline, each Monday was compared to the Monday baseline, etc., to account for differences in the daily POI visit patterns.

4.1 $$\text{per cent change}=100\times \frac{\left(\text{daily value}-\text{baseline value}\right)}{\left(\text{baseline value}\right)}.$$

In equation (4.2), the 7-day rolling averages of these changes were calculated to alleviate daily anomalies and to generate smoother trends. The results presented in the research paper are based on the 7-day rolling average values. Raw values can be found in the electronic supplementary material.

4.2 $$\begin{array}{rl} & 7\text{- day rolling average }\\ & \;=\frac{\text{sum of last 7 daily per}\;\text{cent change values}}{7}. \end{array}$$

### 3.3. Calculating resilience curves and general resilience values

**Figure 4.**
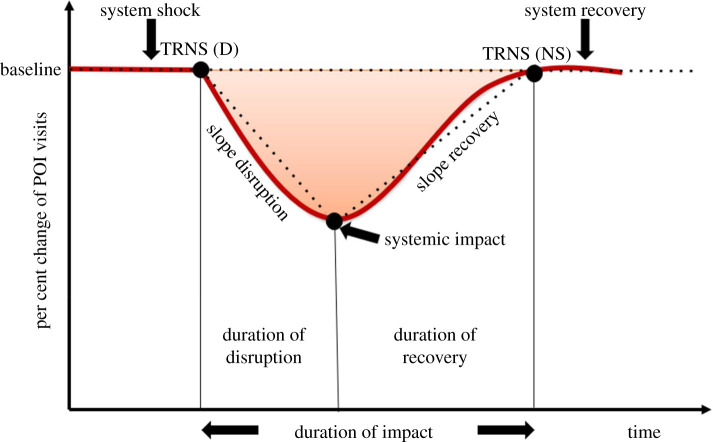
Resilience curve of per cent change of POI visits due to system shock.

Resilience curves (figure 4) were determined for each POI category by plotting the per cent change 7-day rolling averages as our MOP. These curves were confined by the transition point at disruption, TRNS(D), and the transition point post disruption, TRNS(NS), signalling a return to the steady state. For purposes of this study, both values were taken at the baseline level of a 0% change in POI visits from the baseline with a ±2% threshold. This means that the disruption and new steady state points were identified as the first 7-day rolling average values to fall within that range during the disruption and recovery periods. Additionally, the 7-day values had to stay within ±2% of 0 for at least 2 days in a row to confirm the transition point had occurred. Upon the creation of these curves, the systemic impact and duration of impact for each category was determined. These two metrics, referred to in this paper as resilience metrics, are defined as follows:

— **Systemic impact**, originally defined by Vugrin *et al*. [63], represents ‘the difference between a targeted system performance level and an actual system performance following a disruptive event.’ In the case of this study, the systemic impact values were taken as the lowest per cent change values achieved by each category post-Hurricane Harvey landfall.
— **Duration of impact** is the sum of the duration of disruption and duration of recovery for each POI category (i.e. the number of days taken for the daily per cent change value to return to zero post disruption). This variable is the period from TRNS(D) to TRNS(NS).

Additionally, this research modified and employed the GR metric originally developed by Nan & Sansavini [64] incorporating it with the systemic impact and duration of impact. This GR metric is an integrated value that quantifies resilience by considering a systemic impact, slope ratio, recovery ability and time-averaged performance loss based on the resilience curve (figure 4). Since the GR value is ultimately dimensionless and unitless, it only serves to compare the relative resilience of various systems in the same community to the same disruptive event. When discussing GR values for systems affected by the same disruption, a comparatively high GR value indicates a higher level of resilience for that system compared to its counterparts. With this in mind, we used the GR metric to quantify data for community resilience and further compare the resilience of various POI categories in the context of Hurricane Harvey. This GR metric can be calculated for each POI category using equation (4.3):

4.3 $$\begin{array}{rl} \mathrm{GR} & =f(\mathrm{SI},\;\mathrm{SR},\;\mathrm{TAPL},\;\mathrm{RA})\\ & =(100\%+\mathrm{SI})\times \mathrm{SR}\times {\left(\mathrm{TAPL}\right)}^{-1}\times \mathrm{RA}. \end{array}$$

— **Systemic impact (SI)** is as defined above. To calculate the GR metric, however, 100% + SI was used. Since systemic impact values are negative by nature, this process ensures that systemic impact affects only the GR value's magnitude and leaves the sign unaffected and that a higher systemic impact value translates to a lower GR metric and vice versa.
— **Slope ratio (SR**) is the slope of the recovery phase divided by the slope of the disruption phase. The slope of the recovery phase is the slope from the systemic impact to the TRNS(NS) value. The slope of the disruption phase is the slope from the TRNS(D) value to the systemic impact (figure 4). A higher slope ratio value indicates a quicker recovery compared to the level of disruption and its associated rapidity.
— **Time average performance loss (TAPL)** is the ratio of the area between the baseline level and the resilience curve and the duration of impact. As stated by Cortés & Strahan [58], this ratio ‘[incorporates] the effects of total performance loss during disruptive and recovery phases’.
— **Recovery ability (RA)** accounts for the extent to which the new steady state achieved upon recovery compares to the steady state before the system shock. It is essentially the ratio between the initial steady state and final steady state. In this research, since the steady states were both taken as the 0% change baseline, this value is equal to 1 for all categories.

This GR metric considers that the slope of recovery and recovery ability have positive effects on resilience and thus are directly related to the GR metric. Conversely, the magnitude of SI, slope of the disruption phase, and TAPL have a negative effect on resilience and thus are inversely related to the GR metric. For instance, a system with a low systemic impact magnitude and small slope of disruption will have a comparatively high GR value; thus, it will have a greater resilience as it is less vulnerable and could recover faster from the disruptive event. Similarly, when a system has a small TAPL and high slope of recovery, it ‘is more capable of reducing the magnitude and duration of deviation of its performance level from the original and new steady states' [64]. As such, that system will also have a comparatively high GR value and thus be considered more resilient as well. It is worth noting that the GR metric itself incorporates a value called the recovery ability (RA). This value accounts for the possibility that the system reaches a post-disruption steady state that is different from the original. If this new level is higher, the RA value will be greater than 1, corresponding to a higher GR metric. When the original and new steady states are equal, the resulting RA value will be 1. Finally, if the new steady state is lower than the original, the RA value will be less than 1, corresponding to a lower final GR metric. As stated previously, the original and new steady state levels in this research were both taken as a 0% change from the baseline. As such, this recovery ability aspect had no effect on the resulting GR values in this instance.

### 3.4. Calculating the spatial distribution of resilience metrics according to POI visits

To understand the spatial distribution of the resilience metrics, we calculated Moran's I statistic, which is a measure of spatial autocorrelation that identifies correlations among neighbours (census tracts) based on the systemic impact and duration of impact. The ‘pseudo’ *p*-values were calculated using 999 permutations of the spatial analysis with values being statistically significant at *p* < 0.05.

## 4. Results for per cent change from the baseline

**Figure 5.**
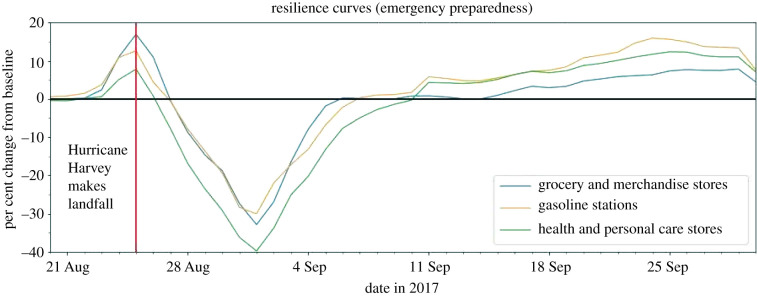
Resilience curves for POIs essential for emergency preparedness.

The 7-day rolling average was used to measure the resilience metrics to minimize the influence of random noise and fluctuations in the POI visits. The time series of data represented by figure 5 through 12 begins 20 August 2017. Prior weeks' data were used to calculate each category's baseline. As such, this region of each graph is relatively flat and near zero. This holds true for all categories except education, for which the last three weeks of October 2017 were used for the baseline. Additionally, 4 September 2017, was Labour Day. All non-essential government offices are closed on Labour Day, and many businesses are also closed or have modified hours. This day fell during the recovery phase of most categories, including those that are mostly government-owned and -operated, such as the US Postal Service and educational facilities. This circumstance thus affects certain categories more than others and would directly increase their duration of impact.

### 4.1. POIs essential for emergency preparedness

**Figure 6.**
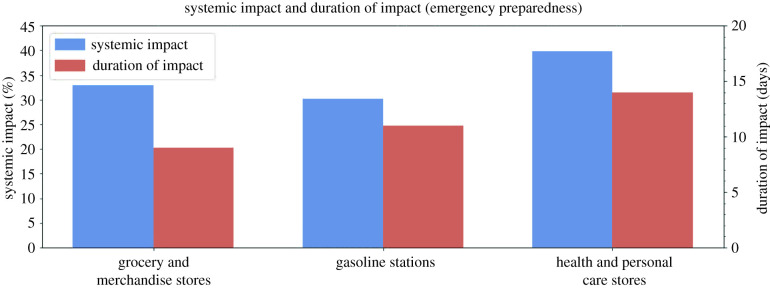
Systemic impact and duration of impact for POIs essential for emergency preparedness.

The group of POIs essential for emergency preparedness captures the various protective actions taken by residents to prepare for disasters. The POI categories essential for emergency preparedness include (1) grocery and merchandise stores, (2) gasoline stations, and (3) health and personal care stores. As shown in figure 5, dramatic increases are evident in the per cent of daily visits for all emergency preparedness categories immediately before Hurricane Harvey made landfall. This implies that residents were filling motor vehicle gas tanks and stockpiling groceries and personal products as preparedness measures. Such increases could be considered measurable shocks to these categories. Compared to the other POI groups, emergency preparedness categories generally experienced GR values on the higher end of the spectrum (figure 5) and a low systemic impact, short duration of impact (figure 6).

#### 4.1.1. Grocery and merchandise

The grocery store category is inclusive of any independent food or meat markets, as well as larger chain stores, such as Kroger, Aldi and Texas' HEB grocery stores. Merchandise stores include businesses such as Walmart and discount stores such as Family Dollar. According to the resilience curve, there was a sharp increase of 16.91% in the 7-day rolling average of visits shortly before the hurricane made landfall. This increase demonstrates residents making last-minute preparations in the form of stockpiling food and other essential supplies. This category saw a systemic impact of −32.87%, a duration of impact of 9 days and a GR value of 4.88.

#### 4.1.2. Gasoline stations

According to figure 5, gasoline station visits experienced an increase of 12.59% shortly before Hurricane Harvey made landfall. This result indicates that Houston residents could be topping up gas tanks of vehicles in preparation for the incoming hurricane. According to the resilience curve, gasoline stations had a systemic impact of a −30.06%, a duration of impact of 11 days, and a GR value of 3.99.

#### 4.1.3. Health and personal care stores

Health and personal care stores include Walgreens, CVS and local pharmacies. These businesses sell personal supplies (also found in grocery and merchandise stores), as well as medical remedies, such as over-the-counter and prescription medication. According to the resilience curve, this category experienced a peak increase of 7.81% on the day the hurricane made landfall, although this was not as significant as the other two POIs essential for emergency preparedness. This 7-day rolling average demonstrates the category's systemic impact of −39.77%, a duration of impact of 14 days and a GR value of 2.42.

### 4.2. POIs essential for emergency response

**Figure 7.**
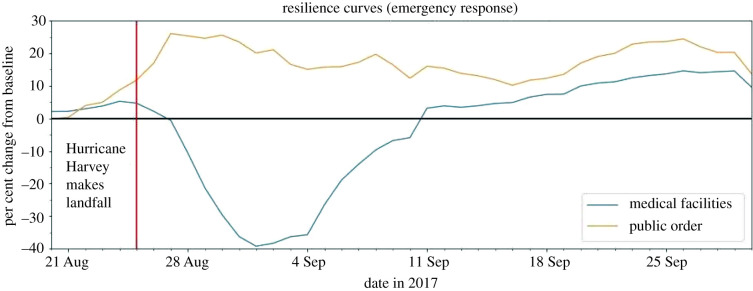
Resilience curves for POIs essential for emergency response.

**Figure 8.**
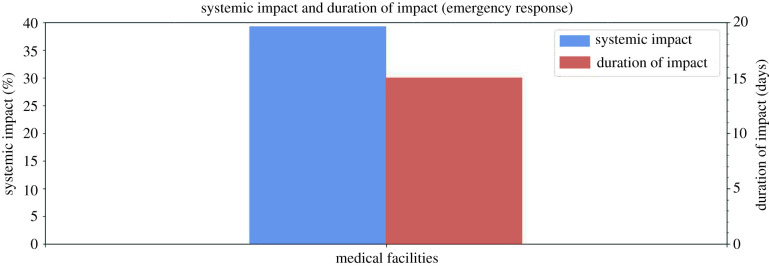
Systemic impact and duration of impact for POIs essential for emergency response.

The POI categories essential for emergency response provide medical and safety services to the community and are most responsive during and in the immediate aftermath of a disaster. This group includes (1) medical facilities and (2) public order. Though grouped together, these services showed very different resilience curves (figure 7) along with different systematic values and duration of impact (figure 8).

#### 4.2.1. Medical facilities

Medical facilities comprise offices of physicians and general medical and surgical hospitals. The resilience curve had a systemic impact of −39.29%, a duration of impact of 15 days and a GR value of 1.39. Though medical facilities had an average systemic impact and duration of impact compared to other categories, it had among the lowest GR values. This could indicate that people did not visit the physical locations of regular medical centres to seek medical support in great numbers, and instead may have visited other temporarily available medical centres.

#### 4.2.2. Public order

Public order consists of any justice, law enforcement, or safety activity, such as county sheriff departments, fire departments and police departments. Unlike every other category, public order did not experience a negative per cent change during Hurricane Harvey. Instead, the category had a staggering increase in the resilience curve, reaching 26.05% increase 2 days after Hurricane Harvey made landfall. These well-above-baseline levels persisted until the beginning of October. This result could be due to the increased requests for emergency response and public order as residents sought relief from the storm and floodwaters. Often, temporary emergency facilities, such as those set up by Federal Emergency Management Agency (FEMA) and other organizations, would collaborate with and work alongside local emergency responders to meet the immediate safety, shelter and medical needs of the community. Since there was no period of negative disruption, the systemic impact, duration of impact and the GR value were not applicable to this POI category.

### 4.3. POIs essential for the recovery activity

**Figure 9.**
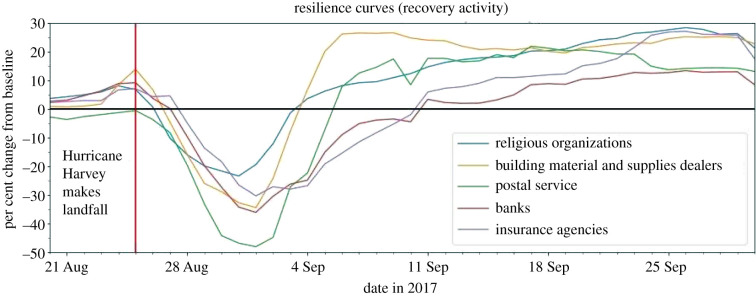
Resilience curves for POIs essential for recovery activity.

**Figure 10.**
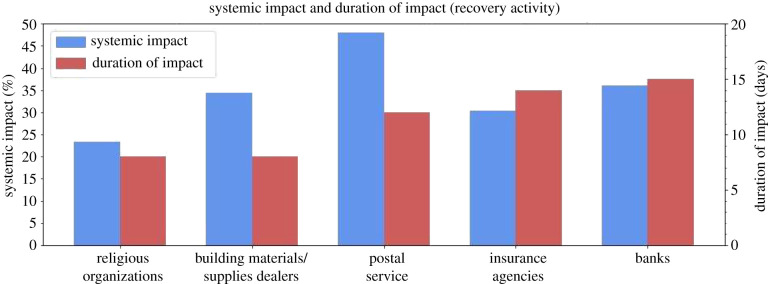
Systemic impact and duration of impact for POIs essential for recovery activity.

The POIs essential for the recovery activity group focuses on both the short-term and long-term recovery from the natural hazards, such as rebuilding homes and managing finances. This group includes (1) religious organizations, (2) building material and supplies dealers, (3) postal service, (4) banks and (5) insurance agencies. Several of these categories experienced slight hikes in visitation just before hurricane impact (figure 9). The only truly notable hike, however, was in the building materials and supplies dealer category, with the others remaining below 10%. The systemic impact and duration of impact values varied for each category (figure 10). All categories in this group experienced sustained, above-baseline levels of visitation throughout September, with levels hovering between 10% and 30% for every category. These numbers did not begin to return to baseline levels until the end of September and the beginning of October.

#### 4.3.1. Religious organizations

The religious organizations category includes houses of worship and other religious and spiritual spaces. The resilience curve displays a systemic impact of −23.38%, a duration of impact of 8 days, and an overall GR value of 8.33. This category also saw a steady increase in visits post-recovery, reaching nearly 30% above the baseline by the end of September. Although the intention of the visits cannot be confirmed, religious organizations are often used to house displaced residents and give out resources to those in need. Additionally, religious centres are nearly omnipresent in local communities and neighbourhoods and many citizens rely heavily upon them during difficult times. This could explain the comparatively low systemic impact and low duration of impact which equate to the highest total GR value of any category.

#### 4.3.2. Building material and supplies dealers

The building material and supplies category includes independent hardware and construction material stores, as well as large chain stores such as Home Depot, Lowe's, and Ace Hardware. The resilience curve depicts a systemic impact of −34.43%, a duration of impact of 8 days, and a GR value of 5.19. Building material and supplies had the second highest GR value of any category, suggesting the immediate reliance on these facilities for recovery efforts. Also, this category's 7-day rolling average hiked to 13.9% on the day as the hurricane made landfall, signalling that large numbers of residents were visiting these locations to gather materials to prepare and to protect their assets. Additionally, this category experienced a sustained above-baseline level of visits post-recovery, with the daily values floating between 20% and 30% for the entirety of September. This suggests that numbers of residents were rebuilding and repairing homes and structures well after the Hurricane devastated the area.

#### 4.3.3. Postal service

This consists almost entirely of United States Postal Service facilities. According to the associated resilience curve, the US Postal Service saw a systemic impact of –48.07%, a duration of impact of 12 days, and a GR value of 2.89. Although this category experienced the highest systemic impact of all the recovery activity categories, it also had a lower duration of impact. The US Postal Service was therefore able to quickly recover from its high impact, leading to a GR value on the higher end of the spectrum compared to all other categories.

#### 4.3.4. Insurance agencies

Insurance agencies refer to smaller, independent businesses as well as to larger corporations, such as Allstate Insurance and State Farm. The resilience curve shows a systemic impact of −30.34%, a duration of impact of 13 days, and a GR value of 2.01. Though the GR value is average, this value does not account for the increased need displayed on the resilience curve throughout September. This sustained above-baseline level suggests residents could be visiting insurance companies to place flood insurance claims, which demonstrates a need to return to a state of normalcy. Residents could also be opening new insurance accounts to be better prepared for future disasters.

#### 4.3.5. Banks

Banks include larger nationwide banks such as Bank of America and Chase, as well as smaller local banks. As displayed in the resilience curve, banks had a systemic impact of −36.09%, a duration of impact of 15 days and a GR value of 1.94. Banks had an average systemic impact and duration of impact along with a slightly lower than average GR value. This could indicate that residents may not immediately need to attend a physical bank to access their finances after a natural hazard such as a hurricane and instead may rely more on online services.

### 4.4. POIs essential for lifestyle and well-being

**Figure 11.**
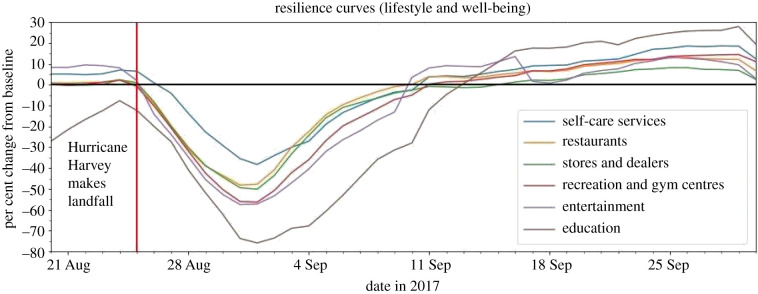
Resilience curves for POIs essential for lifestyle and well-being.

**Figure 12.**
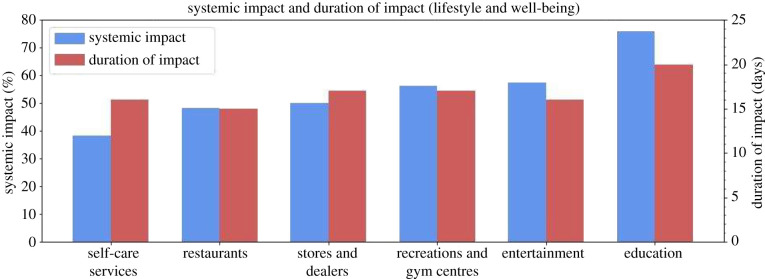
Systemic impact and duration of impact for POIs essential for lifestyle and well-being.

The POIs for the lifestyle and well-being group represent the economic and physical and emotional well-being of residents. The group includes: (1) self-care services, (2) stores and dealers, (3) restaurants, (4) recreation and gym centres, (5) entertainment and (6) education. This group did not experience clear, significant hikes in visitation just before hurricane impact, and the slightly increased levels before impact in certain categories are due simply to variations and inherent error in the baseline (figure 11). This suggests that residents did not prioritize visiting these locations before the disaster. Additionally, these categories often experienced the greatest systemic impact and longest duration of impact (figure 12) compared to other POIs. Although the exact reasons for this mobility pattern cannot be confirmed using only the POI data, the quantification of these visits and previous knowledge in the disaster research implies that these services were not essential to the immediate recovery of the individual household.

#### 4.4.1. Self-care services

Self-care services consist of beauty salons and studios, barbershops, and dentist offices, which address personal care that is not as immediate or imperative as medical services. The resilience curve depicts a systemic impact of −38.29% (figure 11), a duration of impact of 16 days (figure 12), and a GR value of 2.07. While this is the highest GR value for any lifestyle and well-being category, it is roughly in the middle of the pack compared to all other categories.

#### 4.4.2. Restaurants

This group refers to chain restaurants, bars and beverage establishments. The resilience curve had a systemic impact of −48.17% (figure 11), a duration of impact of 15 days (figure 12) and a GR value of 1.85.

#### 4.4.3. Stores and dealers

The stores and dealers category includes but is not limited to clothing, sporting goods, book, electronics and liquor stores, and also includes lessors of real estate, such as storage facilities and shopping centres. The resilience curve displays a systemic impact of –50.14% (figure 11), a duration of impact of 17 days (figure 12) and a GR value of 1.52. While this category's resilience curve closely matches that of restaurants', its GR value is lower mainly because it took 2 more days to recover to the baseline level than did restaurants.

#### 4.4.4. Recreation and gym centres

This group refers to gyms, country clubs, golf courses, and other sports and fitness-related centres. The resilience curve had a systemic impact of −56.35%, a duration of impact of 17 days and a GR value of 1.08.

#### 4.4.5. Entertainment

Entertainment includes locations such as museums, historical sites, and movie theatres, which are associated with places of leisure and social gatherings. The resilience curve depicts a systemic impact of −57.54% (figure 11), a duration of impact of 16 days (figure 12), and a GR value of 0.76. This is the second lowest GR value of all and is largely since its time-averaged performance loss was one of the highest of all.

#### 4.4.6. Education

Education consists of primary schools, secondary schools and colleges. The resilience curve displays a systemic impact of −75.94% (figure 11), a duration of impact of 20 days (figure 12), and a GR value of 0.38. Education had the greatest systemic impact and longest duration of impact, resulting in the lowest GR value of any category. This could be since community residents were more concerned with meeting their immediate needs, such as water, food and shelter, rather than education. Education facilities are also large and require immense effort and care to restore.

**Figure 13.**
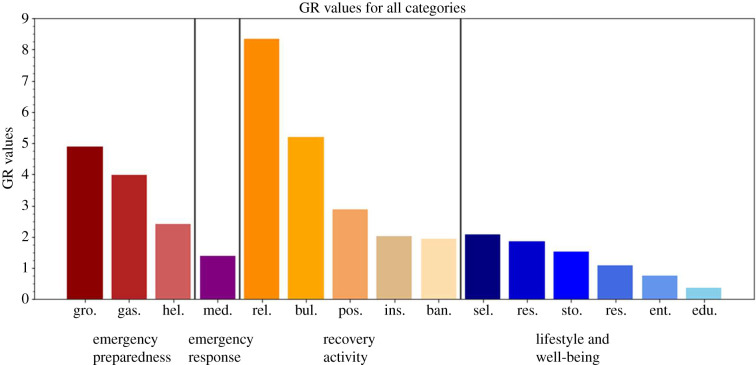
GR metric values for all categories by group.

Figure 13 depicts all GR metric values for every category in this research sorted from highest to lowest GR value in disaster stage group. Once again, the GR value magnitudes are unitless and are mainly used for comparison between two systems impacted by the same event. Moreover, a category with a higher GR value is considered to have a higher resilience than one with a lower value. By this definition, it is clear from this figure that emergency preparedness and recovery activity had the most resilient POI categories. In particular, religious organizations, building material and supplies dealers, grocery and merchandise stores, and gasoline stations were the most resilient categories, suggesting how imperative these POIs are in a disaster and recovery setting. Lifestyle and well-being categories were among the lowest of all, which could demonstrate either the community's lack of demand or lack of accessibility for these POIs during Hurricane Harvey.

## 5. Results for spatial analysis of resilience metrics

**Figure 14.**
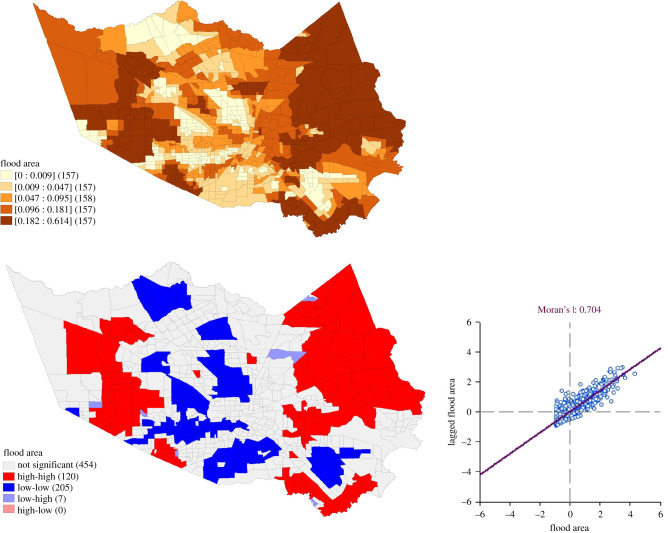
Spatial distribution of flooding and non-flooding inundation areas caused by Hurricane Harvey.

The study accessed flooding data publicly available on the FEMA website. The quantile map of Harris County displays flooding inundation areas divided by the total area of individual census tracts as calculated by spatial rate (figure 14). The distribution of flooding in the second map shows the Moran's I statistic of 0.704 supporting the existence of strong clusters of flooding and non-flooding inundation areas in Harris County, within which the Houston metropolitan is located.

The results are based on Hurricane Harvey's impact on the Houston metropolitan on 28 August 2017, since this day had the greatest number of POI visit drops according to the raw daily visit data. The research also performed spatial analysis using Moran's I statistic correlations and pseudo *p*-values, which are statistically significant at *p* < 0.05, for the systemic impact and duration of impact. Overall, resilience metrics according to POI visits did not show strong evidence of clustering. As shown in table 2, the majority of correlation values are relatively low, indicating a lack of spatial clustering. Nevertheless, grocery and merchandise stores, building material and supplies dealers, entertainment, recreation and gym centres, restaurants, and stores and dealers had low correlations at statistically significant levels for both resilience metrics. Gasoline stations were statistically significant for systemic impact, and education facilities were statistically significant for duration of impact.

**Table 2.** Moran's I statistic correlations and pseudo *p*-values of POI groups. The asterisk (*) signifies a *p*-value less than 0.05 significance level.

POI group	category	systemic impact		duration of impact	
		Moran's I	*p*-value	Moran's I	*p*-value
emergency preparedness	grocery and merchandize stores	0.075	0.0020*	0.061	0.009*
	gasoline stations	0.061	0.027*	0.027	0.143
	health and personal care stores	−0.012	0.402	−0.044	0.159
emergency activity	medical facilities	0.016	0.328	0.056	0.067
recovery activity	religious organizations	0.022	0.200	0.034	0.118
	building material and supplies dealers	0.143	0.001*	0.089	0.016*
	insurance agencies	0.020	0.271	−0.026	0.269
	postal service	−0.406	0.108	−0.487	0.027*
	banks	0.058	0.055	−0.010	0.434
lifestyle and well-being	self-care services	0.011	0.290	0.007	0.361
	restaurants	0.114	0.001*	0.059	0.017*
	stores and dealers	0.046	0.032*	0.037	0.050*
	recreation and gym centres	0.144	0.002*	0.118	0.005*
	entertainment	0.095	0.004*	0.069	0.023*
	education	0.024	0.163	0.064	0.020*

**Figure 15.**
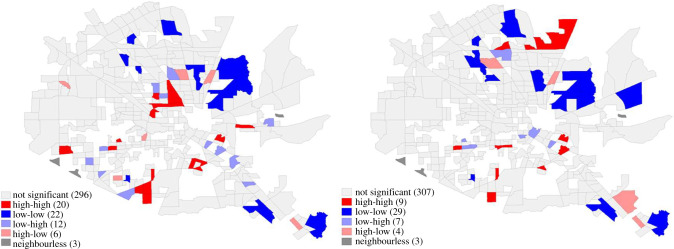
Spatial distribution of systemic impact and duration of impact.

As an example of visual clustering, figure 15 shows the results for resilience metrics of building materials and supplies dealers. The systemic impact and total recovery effort of all census tracts had a fairly strong and statistically significant relationship with a Spearman correlation of 0.89. In other words, high systemic impact appears with long duration of impact while low systemic impact appears with short duration of impact. Using Moran's I statistic, statistically significant (*p* < 0.05) areas of high systemic impact and long duration of impact are highlighted as red clusters while statistically significant areas of low systemic impact and short duration of impact are highlighted as blue clusters. Although the clustering coefficients are low (0.143 for systemic impact and 0.089 for duration of impact, respectively), such values are statistically significant and not by chance. In some instances, clustering overlaps with the flooding inundation data, which may connect the increased exposure to flooding with the lower resilience according to POI visits. However, this relationship does not always hold true. For example, the north eastern blue area has low levels of systemic impact and short duration of impact, although it overlaps with an area of extensive flooding area.

## 6. Discussion and conclusion

This research uses location-intelligence data related to visits to points-of-interest as indicators of the state of complex systems for resilience assessment within communities. Natural hazards cause perturbations to complex systems, such as the residents, built environment and state of the businesses. The aggregated measure of visits to POIs enables understanding of the interaction among these components of complex systems by quantifying the unexpected shocks to essential community facilities and services. The research establishes an inclusive approach using available location-intelligence data, of which researchers, planners and emergency planners can use to examine community resilience. This research applies methodological frameworks to the analysis of variations of POI visits before and after the landfall of Hurricane Harvey in Houston in 2017. The framework quantifies the metrics of systemic impact, duration of impact and the GR value of the community systems based on four POI groups: emergency preparedness, emergency response, recovery activity, and lifestyle and well-being.

Given the novelty of this type of location-intelligence data in academic research, it is worth noting possible limitations of applications of the dataset and guidelines for future use of the data. As with other forms of data collection, a limitation of POI data is that the demographics in location-intelligence data may not be representative of an affected population. Location-intelligence data are limited to those who own electronic devices, such as cell phones [62,65]. The second limitation is the selection of the baseline. It is important to consider the dynamic mobility patterns of a system to establish an accurate baseline, which is used for the comparison with the state of the community after the disruption of the system. Since this research investigated systems at a community level, it established a return to normalcy as reaching pre-disaster conditions of POI visits [63,64]. This assumption was validated as all POI categories returned to baseline levels. Another limitation could be that external factors not related to the disaster impact, such as major community events or celebrations, influenced the data and mobility patterns. To mitigate this limitation, the 7-day rolling average was used to calculate the systemic impact, duration of impact, and ultimately the final GR calculations and spatial distributions.

Finally, it is important to clarify the meaning behind the resilience metrics derived from POI visits data. We cannot fully know if a decrease in the number of visits to a POI is due to a lack of accessibility (e.g. flooded roads), damage to the facility, or lack of interest and demand by the general public. The resilience quantification based on POI visits can be used in conjunction with other sources of data. It is a proxy for a conglomeration of factors enabling understanding and identification of potential areas of vulnerability within a community. Supportable inferences about resilience may be obtained based on an understanding of community resilience and locations most affected by flooding. Nevertheless, these findings allow disaster response planners and emergency responders to visualize the intensity and period of disruption experienced by systems within their community, allowing them to better prioritize response and restoration efforts. In particular, community leaders and emergency planners should take notice of POI categories with the highest and lowest GR values and those which demonstrate spatial clustering. This is due to the holistic nature of POI visits which encapsulate elements of the built environment, impacts of natural hazards, and disruptions to essential services to measure community resilience.

The calculation and analysis of the POI visits data along with previous literature understanding of how such services are integral to the recovery and functionality of the community can reveal unique sights into the quantification of community resilience. Religious organizations, building material and supplies dealers, and grocery and merchandise stores had the highest GR values of those studied. Religious organizations tend to have immense and widespread participation and are also deeply ingrained within their communities, and these are potentially the main forces behind its high resilience. Building material and supplies dealers and grocery and merchandise stores could be indirectly involved in community recovery as they cater to the core basic and safety needs by providing food, supplies and shelter. Furthermore, these two categories had statistically significant clustering of the systemic impact and impact of duration values, which could demonstrate a need to investigate the underlying mechanisms for such groupings. In particular, the visual comparison of statistically significant clusters of the flooding inundation map with the building material and supplies dealers suggest flood exposure is associated with the lower resilience metrics according to POI visits, but at other times, these clusters do not overlap, bringing into question what underlying factors contribute to the clusters of lower resilience metrics. All categories in the lifestyle and well-being group had among the lowest GR values, with recreation and gym centres, entertainment and education being ranked among categories with the lowest scores. These categories also have statistically significant clustering at low correlations. These do not always overlap with areas of extensive flooding, which brings into question the cause of fewer visits. One possibility is that residents experienced a decreased demand for such location visits until after the restoration of basic community and personal functions. Additionally, education had the lowest of all GR values, which could align with the fact that some schools were closed for extended periods or were inaccessible after the hurricane as the community rebounds from the perturbation and school officials attempt to reorganize for reopening.

Despite these inferences, it is important to recognize that the exact cause of the drops in POI visits cannot be determined purely from the POI visits data, rather it is used as a tool to detect possible areas of vulnerability and improvement in systems. Community leaders and emergency planners could use POI categories as proxies to guide resource allocations. The decision-making might entail whether to allocate more resources toward ensuring the essential services with the highest GR values remain functional or rather to focus on the lower GR value categories to improve overall resilience metrics for the POI categories. By comparing the systemic impact, duration of impact and GR values, this research may provide resilience metrics according to POI visits for future applications. Spatial visualizations show census tracts of high and low disaster impact which could not be captured solely from flooding maps. For example, some areas of high disaster impact would not overlap with the areas of frequent flooding. Hence, this study illustrates that using digital trace data—POI visits—complements the current resilience assessment approaches and helps provide a broader view of the interacting complex systems of the communities.

## Data accessibility

Owing to Safegraph's policy on their points-of-interest (POI) data, the authors cannot publicly share the raw dataset used in this study. Researchers interested in the raw POI data should contact SafeGraph at https://www.safegraph.com/, where requests can be made for the data.

## References

[1] Li QC, Dong SJ, Mostafavi A. 2019 Modeling of inter-organizational coordination dynamics in resilience planning of infrastructure systems: a multilayer network simulation framework. PLoS ONE **14**, e0224522. (10.1371/journal.pone.0224522) 31721810 PMC6853286

[2] de Almeida BA, Mostafavi A. 2016 Resilience of infrastructure systems to sea-level rise in coastal areas: impacts, adaptation measures, and implementation challenges. Sustainability-Basel **8**, 1115. (10.3390/su8111115)

[3] Mostafavi A, Ganapati NE, Nazarnia H, Pradhananga N, Khanal R. 2018 Adaptive capacity under chronic stressors: assessment of water infrastructure resilience in 2015 Nepalese earthquake using a system approach. Nat. Hazards Rev. **19**. (10.1061/(Asce)Nh.1527-6996.0000263)

[4] Peacock WG, Ragsdale K. 1997 Social systems, ecological networks, and disasters: toward a socio-political ecology of disasters. In Hurricane Andrew: ethnicity, gender and the sociology of disaster (eds WG Peacock, BH Morrow, H Gladwin), pp. 20-35. London, UK: Routledge.

[5] Rasoulkhani K, Mostafavi A, Reyes MP, Batouli M. 2020 Resilience planning in hazards–humans–infrastructure nexus: a multi-agent simulation for exploratory assessment of coastal water supply infrastructure adaptation to sea-level rise. Environ. Modell. Softw. **125**, 104636. (10.1016/j.envsoft.2020.104636)

[6] Pattel SS, Rogers MB, Amlot R, Rubin JG. 2017 What do we mean by 'community resilience'? A systematic literature review of how it is defined in the literature. PLoS Curr. Disasters **9**. (10.1371/currents.dis.db775aff25efc5ac4f0660ad9c9f7db2) PMC5693357 29188132

[7] Ramirez-Marquez JE, Rocco CM, Barker K, Moronta J. 2018 Quantifying the resilience of community structures in networks. Reliab. Eng. Syst. Safe **169**, 466-474. (10.1016/j.ress.2017.09.019)

[8] Yu JZ, Baroud H. 2019 Quantifying community resilience using hierarchical bayesian kernel methods: a case study on recovery from power outages. Risk Anal. **39**, 1930-1948. (10.1111/risa.13343) 31287575

[9] Dong SJ, Wang HZ, Mostafavi A, Gao JX. 2019 Robust component: a robustness measure that incorporates access to critical facilities under disruptions. J. R Soc. Interface **16**, 20190149. (10.1098/rsif.2019.0149) 31387488 PMC6731514

[10] Davis CA, Mostafavi A, Wang HZ. 2018 Establishing characteristics to operationalize resilience for lifeline systems. Nat. Hazards Rev. **19**. (10.1061/(Asce)Nh.1527-6996.0000303)

[11] Applied Technology Council. 2016 Critical assessment of lifeline system performance: understanding societal needs in disaster recovery. NIST GCR 16-917-39. Gaithersburg, MD: U.S. Department of Commerce National Institute of Standards and Technology Engineering Laboratory. (10.6028/nist.Gcr.16-917-39)

[12] Batouli M, Mostafavi A. 2018 Multiagent simulation for complex adaptive modeling of road infrastructure resilience to sea-level rise. Comput-Aided Civ. Inf. **33**, 393-410. (10.1111/mice.12348)

[13] Guikema S, Nateghi R. 2018 Modeling power outage risk from natural hazards. In Oxford research encyclopedia of natural hazard science. Oxford, UK: Oxford University Press. (10.1093/acrefore/9780199389407.013.52)

[14] Rourke TD. 2007 Critical infrastructure, interdependencies, and resilience. Bridge Linking Eng. Soc. **37**, 22-29.

[15] Cimellaro GP, Reinhorn AM, Bruneau M. 2010 Seismic resilience of a hospital system. Struct. Infrastruct. E **6**, 127-144. (10.1080/15732470802663847)

[16] Zhong S, Clark M, Hou XY, Zang YL, Fitzgerald G. 2014 Development of hospital disaster resilience: conceptual framework and potential measurement. Emerg. Med. J. **31**, 930. (10.1136/emermed-2012-202282) 24028975

[17] Baum NL, Stokar YN, Ginat-Frolich R, Ziv Y, Abu-Jufar I, Cardozo BL, Pat-Horenczyk R, Brom D. 2018 Building Resilience Intervention (BRI) with teachers in Bedouin communities: from evidence informed to evidence based. Child Youth Serv. Rev. **87**, 186-191. (10.1016/j.childyouth.2018.02.036)

[18] Aghababaei M, Koliou M, Watson M, Xiao Y. 2020 Quantifying post-disaster business recovery through Bayesian methods. Struct. Infrastruct. E (10.1080/15732479.2020.1777569)

[19] Middleton EJ.T, Latty T. 2016 Resilience in social insect infrastructure systems. J. R Soc. Interface **13**, 20151022. (10.1098/rsif.2015.1022) 26962030 PMC4843670

[20] Comes T, Van de Walle B. 2014 Measuring disaster resilience the impact of hurricane sandy on critical infrastructure systems. In Proc. of the 11th Int. ISCRAM Conf., University Park, PA, May, pp. 190-199. See http://idl.iscram.org/files/comes/2014/408_Comes+VanDeWalle2014.pdf.

[21] Singh-Peterson L, Lawrence G. 2015 Insights into community vulnerability and resilience following natural disasters: perspectives with food retailers in Northern NSW, Australia. Local Environ. **20**, 782-795. (10.1080/13549839.2013.873396).

[22] Spiegler VLM, Potter AT, Naim MM, Towill DR. 2016 The value of nonlinear control theory in investigating the underlying dynamics and resilience of a grocery supply chain. Int. J. Prod. Res. **54**, 265-286. (10.1080/00207543.2015.1076945)

[23] Esmalian A, Dong S, Coleman N, Mostafavi A. In press. Determinants of risk disparities due to infrastructure service losses in disasters: a household service gap model. Risk Anal. (10.1111/risa.13738) 33914344

[24] Mostafavi A, Ganapati NE. 2019 Toward convergence disaster research: building integrative theories using simulation. Risk Anal. (10.1111/risa.13303) 30884546

[25] Weilant S, Strong A, Miller BM. 2019 Incorporating resilience into transportation planning and assessment. Santa Monica, CA: RAND Corporation. See https://www.rand.org/pubs/research_reports/RR3038.html.

[26] Dong SJ, Esmalian A, Farahmand H, Mostafavi A. 2020 An integrated physical-social analysis of disrupted access to critical facilities and community service-loss tolerance in urban flooding. Comput. Environ. Urban **80**, e10144310. (doi:1016/j.compenvurbsys.2019.101443)

[27] Coleman N, Esmalian A, Mostafavi A. 2020 Anatomy of susceptibility for shelter-in-place households facing infrastructure service disruptions caused by natural hazards. Int. J. Disast. Risk Reduct. **50**, 101875. (10.1016/j.ijdrr.2020.101875)

[28] Coleman N, Esmalian A, Mostafavi A. 2020 Equitable resilience in infrastructure systems: empirical assessment of disparities in hardship experiences of vulnerable populations during service disruptions. Nat. Hazards Rev. **21**, 04020034. (10.1061/(Asce)Nh.1527-6996.0000401)

[29] Berlemann M. 2016 Does hurricane risk affect individual well-being? Empirical evidence on the indirect effects of natural disasters. Ecol. Econ. **124**, 99-113. (10.1016/j.ecolecon.2016.01.020)

[30] Morss RE, Mulder KJ, Lazo JK, Demuth JL. 2016 How do people perceive, understand, and anticipate responding to flash flood risks and warnings? Results from a public survey in Boulder, Colorado, USA. J. Hydrol. **541**, 649-664. (10.1016/j.jhydrol.2015.11.047)

[31] Sherrieb K, Norris FH, Galea S. 2010 Measuring capacities for community resilience. Soc. Indic Res. **99**, 227-247. (10.1007/s11205-010-9576-9)

[32] Webb GR, Tierney KJ, Dahlhamer JM. 2002 Predicting long-term business recovery from disaster: a comparison of the Loma Prieta earthquake and Hurricane Andrew. Glob. Environ. Change Part B Environ. Hazards **4**, 45-58. (10.1016/s1464-2867(03)00005-6)

[33] Wang Q, Taylor JE. 2014 Quantifying human mobility perturbation and resilience in hurricane sandy. PLoS ONE **9**, e112608. (10.1371/journal.pone.0112608) 25409009 PMC4237337

[34] Zhai W, Peng ZR, Yuan FX. 2020 Examine the effects of neighborhood equity on disaster situational awareness: Harness machine learning and geotagged Twitter data. Int. J. Disast. Risk Reduct. **48**, 101611. (10.1016/j.ijdrr.2020.101611)

[35] Zhang C, Fan C, Yao WL, Hu X, Mostafavi A. 2019 Social media for intelligent public information and warning in disasters: an interdisciplinary review. Int. J. Inform. Manage **49**, 190-207. (10.1016/j.ijinfomgt.2019.04.004)

[36] Zhang C, Yao WL, Yang Y, Huang RH, Mostafavi A. 2020 Semiautomated social media analytics for sensing societal impacts due to community disruptions during disasters. Comput-Aided Civ. Inf **35**, 1331-1348. (10.1111/mice.12576)

[37] Yuan FX, Liu R, Mao L, Li M. 2021 Internet of people enabled framework for evaluating performance loss and resilience of urban critical infrastructures. Safety Sci. **134**, 105079. (10.1016/j.ssci.2020.105079)

[38] Yuan FX, Li M, Liu R. 2020 Understanding the evolutions of public responses using social media: Hurricane Matthew case study. Int. J. Disast. Risk Reduct. **51**, 101798. (10.1016/j.ijdrr.2020.101798)

[39] Rasoulkhani K, Mostafavi A. 2018 Resilience as an emergent property of human-infrastructure dynamics: a multi-agent simulation model for characterizing regime shifts and tipping point behaviors in infrastructure systems. PLoS ONE **13**, e0207674. (10.1371/journal.pone.0207674) 30462719 PMC6248985

[40] Yuan FX, Liu R. 2020 Mining social media data for rapid damage assessment during hurricane matthew: feasibility study. J. Comput. Civil Eng. **34**. (10.1061/(Asce)Cp.1943-5487.0000877)

[41] Yuan FX, Liu R. 2018 Feasibility study of using crowdsourcing to identify critical affected areas for rapid damage assessment: Hurricane Matthew case study. Int. J. Disast. Risk Reduct. **28**, 758-767. (10.1016/j.ijdrr.2018.02.003)

[42] Yuan FX, Liu R. 2019 Identifying damage-related social media data during hurricane matthew: a machine learning approach. In Computing in civil engineering 2019: visualization, information modeling, and simulation, pp. 207-214.

[43] Fan C, Mostafavi A. 2019 A graph-based method for social sensing of infrastructure disruptions in disasters. Comput-Aided Civ. Inf. **34**, 1055-1070. (10.1111/mice.12457)

[44] Fan C, Esparza M, Dargin J, Wu FS, Oztekin B, Mostafavi A. 2020 Spatial biases in crowdsourced data: social media content attention concentrates on populous areas in disasters. Comput. Environ Urban **83**, 101514. (10.1016/j.compenvurbsys.2020.101514)

[45] Fan C, Zhang C, Yahja A, Mostafavi A. 2021 Disaster City Digital Twin: a vision for integrating artificial and human intelligence for disaster management. Int. J. Inform. Manage **56**, 102049. (10.1016/j.ijinfomgt.2019.102049)

[46] Yabe T, Tsubouchi K, Fujiwara N, Sekimoto Y, Ukkusuri SV. 2020 Understanding post-disaster population recovery patterns. J. R Soc. Interface **17**, 20190532. (10.1098/rsif.2019.0532) 32070218 PMC7061695

[47] Lu X et al. 2016 Unveiling hidden migration and mobility patterns in climate stressed regions: a longitudinal study of six million anonymous mobile phone users in Bangladesh. Glob. Environ. Change **38**, 1-7. (10.1016/j.gloenvcha.2016.02.002)

[48] Yabe T, Zhang YC, Ukkusuri SV. 2020 Quantifying the economic impact of disasters on businesses using human mobility data: a Bayesian causal inference approach. Epj Data Sci. **9**, 36. (10.1140/epjds/s13688-020-00255-6)

[49] National Weather Service, NOAA. 2019 Major hurricane harvey - August 25–29, 2017. See https://www.weather.gov/crp/hurricane_harvey.

[50] Khare A, He Q, Batta R. 2020 Predicting gasoline shortage during disasters using social media. Or Spectrum **42**, 693-726. (10.1007/s00291-019-00559-8)

[51] Beatty TK.M., Shimshack JP, Volpe RJ. 2019 Disaster preparedness and disaster response: evidence from sales of emergency supplies before and after hurricanes. J. Assoc. Environ. Res. **6**, 633-668. (10.1086/703379)

[52] Adams TM, Stewart LD. 2014 Chaos theory and organizational crisis: a theoretical analysis of the challenges faced by the New Orleans Police Department during Hurricane Katrina. Public Organiz. Rev. **15**, 415-431. (10.1007/s11115-014-0284-9)

[53] Murphy H. 2019 Policing in natural disasters: stress, resilience, and the challenges of emergency management. J. Publ. Adm. Res. Theor. **29**, 648-650. (10.1093/jopart/muz008)

[54] FEMA. 2007 Public Assistance Debris Management Guide. See https://www.fema.gov/sites/default/files/2020-07/fema_325_public-assistance-debris-mgmt-plan_Guide_6-1-2007.pdf.

[55] EPA. 2019 Planning for Natural Disaster Debris. See https://www.epa.gov/sites/production/files/2019-05/documents/final_pndd_guidance_0.pdf.

[56] Kousky C. 2017 Disasters as learning experiences or disasters as policy opportunities? Examining flood insurance purchases after hurricanes. Risk Anal. **37**, 517-530. (10.1111/risa.12646) 27576946

[57] Klomp J. 2014 Financial fragility and natural disasters: an empirical analysis. J. Fin. Stab. **13**, 180-192. (10.1016/j.jfs.2014.06.001)

[58] Cortés KR, Strahan PE. 2017 Tracing out capital flows: how financially integrated banks respond to natural disasters. J. Fin. Econ. **125**, 182-199. (10.1016/j.jfineco.2017.04.011)

[59] Stevens N. Postal Service for Katrina. 2005 CRS Report for Congress. University of North Texas Libraries Government Documents Department. See https://www.everycrsreport.com/files/20051102_RS22245_140577d73e61dcec69399f1c1bc087fde88cbbbc.pdf.

[60] Marshall MI, Schrank HL. 2014 Small business disaster recovery: a research framework. Nat. Hazards **72**, 597-616. (10.1007/s11069-013-1025-z)

[61] Corey CM, Deitch EA. 2011 Factors affecting business recovery immediately after Hurricane Katrina. J. Conting. Crisis Manag. **19**, 169-181. (10.1111/j.1468-5973.2011.00642.x)

[62] Dargin JS, Mostafavi A. 2020 Human-centric infrastructure resilience: uncovering well-being risk disparity due to infrastructure disruptions in disasters. PLoS ONE **15**, e0234381. (10.1371/journal.pone.0234381) 32555741 PMC7302446

[63] Vugrin ED, Warren DE, Ehlen MA, Camphouse RC. 2010 A framework for assessing the resilience of infrastructure and economic systems. Sust. Res. Crit. Infrastruct. Syst.: Simul. Model. Intell. Eng., 77-116. (10.1007/978-3-642-11405-2_3)

[64] Nan C, Sansavini G. 2017 A quantitative method for assessing resilience of interdependent infrastructures. Reliab. Eng. Syst. Safe **157**, 35-53. (10.1016/j.ress.2016.08.013)

[65] Wesolowski A, Eagle N, Noor AM, Snow RW, Buckee CO. 2013 The impact of biases in mobile phone ownership on estimates of human mobility. J. R Soc. Interface **10**, 20120986. (10.1098/rsif.2012.0986) 23389897 PMC3627108

